# Into the *Thermus* Mobilome: Presence, Diversity and Recent Activities of Insertion Sequences Across *Thermus* spp.

**DOI:** 10.3390/microorganisms7010025

**Published:** 2019-01-21

**Authors:** Alba Blesa, Mercedes Sánchez, Eva Sacristán-Horcajada, Sandra González-de la Fuente, Ramón Peiró, José Berenguer

**Affiliations:** 1 Department of Biotechnology, Faculty of Experimental Sciences, Universidad Francisco de Vitoria, Madrid 28223, Spain; alba.blesa@ufv.es; 2 Centro de Biología Molecular Severo Ochoa (CBMSO), Universidad Autónoma de Madrid-Consejo Superior de Investigaciones Científicas, Madrid 28049, Spain; mercedes.sanchez@cbm.csic.es (M.S.); esacristan@cbm.csic.es (E.S.-H.); sandra.g@cbm.csic.es (S.G.-d.l.F.); rpeiro@cbm.csic.es (R.P.)

**Keywords:** insertion sequence, transposons, transposases, HGT, *Thermus*, thermophiles, mobilome

## Abstract

A high level of transposon-mediated genome rearrangement is a common trait among microorganisms isolated from thermal environments, probably contributing to the extraordinary genomic plasticity and horizontal gene transfer (HGT) observed in these habitats. In this work, active and inactive insertion sequences (ISs) spanning the sequenced members of the genus *Thermus* were characterized, with special emphasis on three *T. thermophilus* strains: HB27, HB8, and NAR1. A large number of full ISs and fragments derived from different IS families were found, concentrating within megaplasmids present in most isolates. Potentially active ISs were identified through analysis of transposase integrity, and domestication-related transposition events of ISTth7 were identified in laboratory-adapted HB27 derivatives. Many partial copies of ISs appeared throughout the genome, which may serve as specific targets for homologous recombination contributing to genome rearrangement. Moreover, recruitment of IS1000 32 bp segments as spacers for CRISPR sequence was identified, pointing to the adaptability of these elements in the biology of these thermophiles. Further knowledge about the activity and functional diversity of ISs in this genus may contribute to the generation of engineered transposons as new genetic tools, and enrich our understanding of the outstanding plasticity shown by these thermophiles.

## 1. Introduction

The amazing diversity of the prokaryotic world is a result of the countless strategies with which they deal with an unpredictable, ever-changing environment. Indeed, microbes display a great capacity for rapid adaptation, accelerating their evolution via the acquisition of novel DNA through horizontal gene transfer (HGT) mechanisms [1,2,3]. Phylogenetic studies have revealed that molecular machines that confer such adaptive advantages are frequently encoded in mobile genetic elements (MGEs), such as plasmids, integrons, transposons, and phages, among others [4,5]. An MGE can be defined as a DNA segment encoding proteins that mediate the movement of DNA either within a cell or between different cellular genomes [4,6,7]. Intracellular movement of DNA is mainly driven by promiscuous recombining DNA, primarily insertion sequences (ISs). ISs consist of up to 2–5 kb-long DNA segments which only encode the minimum information required to enable their mobility [8], including a transposase, an enzyme that recognizes specific short inverted repeats (IRs) located at the extremes of the ISs, and catalyzes its mobilization into a new site. These insertions may inactivate genes or activate transcription of downstream genes, leading to DNA rearrangements when acting as targets for homologous recombination [8]. As ISs can also “land” within conjugative plasmids, integrative and conjugative elements (ICE), or (pro)phages, they can “ride along” these transferable elements, increasing their dissemination and impact on diverse microbial genomes [9,10,11]. Therefore, there is great interest in the role of these ISs, not only in microbial evolution, but also in the spread and expression of virulence factors, antimicrobial resistance, or biotransformation of xenobiotics in many bacteria [12,13,14,15].

The growing availability of complete genome sequences has increased the number of ISs identified in all domains of life [16], showing a common structure where an IR flanks one or two genes encoding the transposase. The molecular mechanisms that catalyze and control transposition are notably heterogeneous, leading to significant diversity among ISs. In order to set a framework for the systematic classification of ISs, Mahillon and Chandler (1998) established a scheme based on the main features of transposase structure and function, dividing them into families and subfamilies (ISFinder database; www-is.biotoul.fr) [17]. 

Thermophilic environments seem to favor genetic plasticity, with frequent genome rearrangements and DNA movement between phylogenetically unrelated microorganisms, even those from different domains [18,19,20]. This intense gene shuffling is partly orchestrated by MGEs which mediate either intracellular or intercellular DNA mobility, the latter headed by frequent HGT events [4,20,21,22]. In fact, HGT is common among bacteria but especially relevant in thermophilic organisms, where replication burden limits the size of the genomes. For instance, the highly efficient conjugative plasmids of *Sulfolobus* spp. and the extraordinary competence capacity of *Thermus thermophilus* have been proposed as the major drivers of these organisms’ adaptability to unexpected environmental shifts. Indeed, IS propagation is thought to be frequent among prokaryotes and maximal among thermophiles [23]. Complete genome sequences of thermophilic organisms have revealed an outstanding abundance and diversity of ISs, sometimes disproportional to their genome size, which would suggest positive selection [24,25,26]. 

The abundance of truncated ISs scattered throughout the genomes of thermophilic bacteria suggests a high frequency of genomic rearrangement, likely related to the evolutionary history and speciation of these strains, as reported for the alkalo-thermophilic bacteria *Bacillus halodurans* [26] or the cyanobacterial *Synechococcus* spp. isolated from hot springs [25]. Thermophilic archaea are also characterized by intense transpositional activity, with the order Sulfolobales being one of the major representatives of such diversity; *S. neocofandices* has a genome with ISs making up more than 10% of its genome [16,27].

In addition to large genomic rearrangements, homologous recombination between IS copies spread across mobile elements may facilitate acquisition of new DNA sequences. In this way, ISs may be considered to be active contributors to pan-genome systems. 

The genus *Thermus* belongs to the ancient clade *Deinococcus–Thermus* [28,29], having several representatives isolated worldwide from geothermal origins, in self-heating material, such as compost, and industrial heating systems. The metabolic capabilities of these strains are very diverse, despite their small genome size, with some strictly aerobic strains and others with anaerobic respiration capacity based on nitrogen oxides or metals, and different abilities to utilize polysaccharides. This assortment of phenotypic traits is frequently encoded by megaplasmids and, thus, may be laterally transferred by different mechanisms, contributing to the fast ecological adaptation of these bacteria to sudden changes in a sort of shared pan-genome [30].

Sequencing studies have revealed the presence of MGEs in *Thermus* species, in particular, in strains that are regarded as excellent laboratory models [31], allowing the identification of recent transpositional events [1,32]. In this way, a few cases of active ISs have previously been reported in *T. thermophilus*, including IS1000B in strain HB8 [33,34] and ISTth7 in strain HB27 [35]. However, more recent genomic data suggest that a much greater frequency of transpositional events than initially though takes place in the genus [30]. 

In this article, we aim to gain insights into the MGEs present in *Thermus* spp., reporting the diversity and abundance of ISs identified in the published *Thermus* genomes. To further study their activity, we focused on three *T. thermophilus* isolates, including a newly sequenced strain, and analyze recent domestication-related jumps of one of the most abundant ISs of *T. thermophilus* HB27. 

## 2. Materials and Methods

*Strains.* Subcultures of *T. thermophilus* HB27 (DSM7039), used for long periods in different laboratories in Germany (hereafter HB27A) and Spain (HB27E), were analyzed for domestication-related genomic changes. The nitrate respiring strain *T. thermophilus* NAR1 [36] was sequenced de novo. All strains were grown at 65 °C under aerobic conditions (150 rpm rotational shaking) in *Thermus* broth (TB) as described previously [37]. 

*DNA isolation and sequencing.* Erlenmeyer flasks filled with 250 mL pre-warmed TB (1/5 volume) and a 1/1000 dilution of overnight culture were grown until stationary phase (1.2 OD_550nm_). Cells were harvested by centrifugation (10 min, 5000 g) for genomic DNA extraction and purification with the Qiagen DNeasy Blood and Tissue Extraction Kit (cat.no. 69504, Hilden, Germany) following manufacturers’ indications. Purity and concentration of the DNA was checked with a NanoDrop ND-1000 (Thermo Scientific, Wilmington, DE, USA) spectrophotometer, and integrity was checked by electrophoresis in 1% agarose gels.

Genomic DNA from *T. thermophilus* HB27A and HB27E were adjusted to 30 ng/μL in Tris-HCl pH 8.8 and sent to Microbes NG (UK) for whole genome sequencing employing Illumina DNAseq technique. Genomic DNA (20 μg/μL) from *T. thermophilus* NAR1 was sequenced de novo at the Norwegian Sequencing Centre (www.sequencing.uio.no) using Pacific Biosciences technology (PacBio RS II).

*Bioinformatic analysis of T. thermophilus NARI.* A total of 60,929 PacBio SMRT reads were provided by the sequencing center. De novo genome assembly was performed following a hierarchical genome-assembly process [38], using the software HGAP v3 (Pacific Biosciences, SMRT Analysis Software v2.3.0, Menlo Park, CA, USA) and circularization with Minimus2 (using the workflow recommended by Pacific Biosciences at https://github.com/PacificBiosciences/Bioinformatics-Training/wiki/Circularizing-and-trimming College Park, MD, USA) [39]. As a result, the genome was assembled into four contigs, corresponding to the bacterial chromosome, two megaplasmids, and one small plasmid. 

Due to the detection of systematic 1 bp deletions in homopolymeric regions in PacBio reads, the assembly was corrected with Illumina reads combining PacBio-Utilities [40] and Pilon [41]. Sequence deletions were corrected when they were supported by an Illumina coverage higher than 10 and the indel was present in at least 50% of the reads. Through this workflow, a final assembly was obtained, consisting of four contigs of 2,021,843; 370,865; 77,135; and 9799 bp. Lastly, annotation of the final assembly was obtained using PROKKA with default parameters [42]. 

*Nucleotide sequence accession number.* The sequence from *T. thermophilus* NARI genome has been submitted at the European Nucleotide Archive (ENA; http://www.ebi.ac.uk/ena/) with the accession PRJEB29203.

*Bioinformatic analysis of T. thermophilus HB27A and HB27E.* The assembly and annotation files from the Illumina reads of HB27A and HB27E samples were provided by the sequencing center. FASTQC (http://www.bioinformatics.babraham.ac.uk/projects/fastqc/) was applied for quality checking of reads, which were subsequently aligned to the reference genome of *T. thermophilus* HB27 (AE017221.1 and AE017222.1 for the chromosome and megaplasmid, respectively) using BWA aligner [43].

*Identification of ISs*. Identification of ISs in the sequences of *T. thermophilus* strains HB27 (AE017221.1 and AE017222), HB8 (GCA_000091545.1 and AB677526), and NAR1 (PRJEB29203) was carried out at the NGS computing facility of the CBMSO. An in-house script was designed to download the ISFinder database (www-is.biotoul.fr) and locally emulate the ISFinder BLAST service, with additional parameters to personalize the alignment. Next, IS detection was performed across the genomes of *T. thermophilus* HB8, HB27, and NARI, using 80% nucleotide identity by BLASTn and 30% amino acid identity by BLASTX as thresholds to identify IS-associated ORFs. Genes identified and annotated as transposases within other *Thermus* genomes were downloaded from the JGI database (https://img.jgi.doe.gov/) and classified according to their IS family, as above. When required, specialized websites such as ACLAME, MITE-Hunter, RepSeek, Repeat Finder, or Repeat Masker were used. Consensus sequences of *T. thermophilus* IS typologies (*ISTth1* to *8* and *IS1000A/B*) were searched using BLAST against the genomes. Wherever possible, this information was checked with the IS index in the ISFinder database for the different organisms. Multiple alignments of copies belonging to the same *ISTth* family, found within the same strain or in different *Thermus* strains, were performed using MUSCLE [44]. *ISTth* copies found in other bacterial genera were also aligned using this software to check for conserved sequences. 

Putatively active IS copies were identified by comparison of recognized ORFs with the sequence of the corresponding transposase in GenBank.

*Phylogenetic analysis*. For each full-length consensus sequence of *ISTth* reported by the ISfinder, BLASTn analysis was performed either within or excluding the *Thermus* genomes (taxi:270). Nucleotide sequences from the best hits (E-value < 10^−5^) were clustered and a neighbor-joining tree was performed using PHYLIP [45].

*Transposase mobilization in T. thermophilus HB27*. In order to detect recent movement of the ISTth7 transposase, we extracted FASTQ files from the soft-clipped regions (a sequence that may not be aligned from the first residue to the last) of the HB27A and HB27E alignments using the extractSoftclipped8 tool, comparing them against the ISTth7 sequence. With these soft-clipped reads, we performed a SoftClip alignment to extract ISTth7 coordinate positions in HB27A and HB27E.

## 3. Results

### 3.1. Incidence of ISs among Members of the Genus Thermus

**Figure 1:**
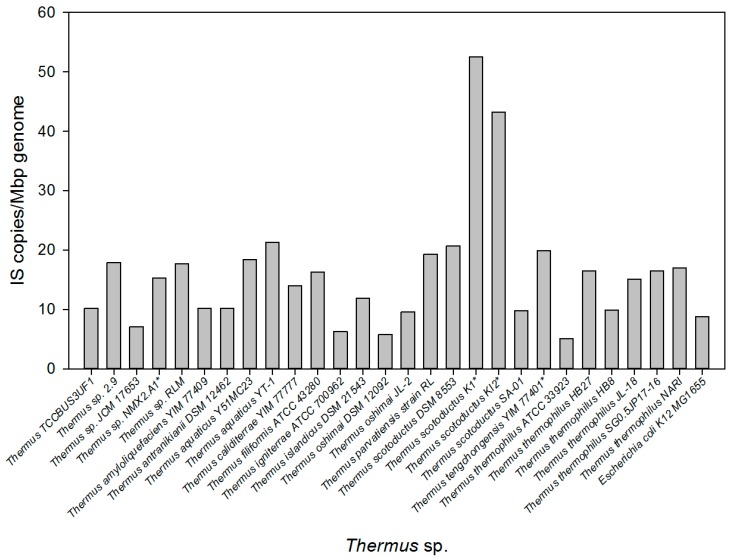
Distribution of insertion sequences (ISs) in *Thermus* spp. Number of genes annotated as transposases were determined in 18 available genomes at NCBI, RefSeq, and JGI. The genome of *T. thermophilus* NAR1 is described in this article. The number of IS copies was normalized to the genome size in Mbp; a strain of *E. coli* was included for comparison.

The availability of the complete genomes of several *Thermus* spp. enabled an exhaustive analysis of IS presence within these thermophiles. Preliminary exploration of genes annotated as transposases across 18 *Thermus* genomes revealed a higher frequency compared to controls, with an average number of 16.21 ± 1.99 IS copies per Mbp, compared with 8.84 in *E. coli* K12 (Figure 1). Notably, more than twice as many hits were found in *T. scotoductus* K1 and KI2 strains, representing more than 4% of its coding sequence. However, the numbers should be cautiously analyzed as data for these strains was extracted from permanent draft genomes available at JGI database, and it is possible that contig boundaries could have been disregarded by the annotation programs. Implementation of an in silico multi-approach looking for IS scars (defined as IS fragments left in the genome as evidence of previous transposition events), IRs, and transposase motifs in the model organisms *T. thermophilus* HB8 and HB27, and in the partial denitrifying strain NAR1, revealed that, in many cases, partial IS copies were more abundant than full copies (Table 1). However, these results include the identification of several tandem copies of a 32 bp-long DNA segment identical to the 5′ extreme of IS1000A/B (positions 1165–1196) in *T. thermophilus* HB8 and HB27 which, after manual analysis, were finally identified as CRISPR repeats separated by 30–40 bp spacers. Actually, 52 copies of this repeated sequence were found in 6 clusters in *T. thermophilus* HB8 and 38 copies divided among 5 clusters in *T. thermophilus* HB27. This same 32 bp DNA sequence also appeared isolated (and, thus, independent of any CRISPR system) in the genome of other *Thermus* spp., suggesting their recruitment for CRISPR-Cas defense in the aforementioned strains. 

### 3.2. Diversity of ISs among Thermus spp. ISTth Families

Not only were ISs abundant within *Thermus* isolates, but they also showed an outstanding diversity (Table 2). As expected, closely related species harbored ISs that were almost identical in sequence, whereas phylogenetically divergent isolates carried IS sequence variants. BLASTn analysis revealed strain-specific ISs in *T. thermophilus* and *T. scotoductus* genomes, whereas a more generalized description was provided for IS4-type (found in *T. thermophilus* SG0.5 and *T. tengchongensis*) and IS30-type sequences (found in *Thermus* TCCBUS3UF1 and *T. oshimai*; data not shown). An exception to this was the conspicuous presence of an IS3-like transposase, frequently found as a full copy termed OrfB in ISFinder. Copies of this IS belonging to the IS605 subfamily were found in *T. aquaticus*, a set of *T. thermophilus* strains such as HB27, JL-18, and SG0.5, and *T. oshimai* strains, showing reasonable conservation (data not shown). 

Despite some strain-specific traits, common aspects could be established regarding the copies found in the model organism *T. thermophilus* (reviewed in [8]) and available in the ISfinder database. The ISs were classified into typologies by features, including similarity of their terminal IR sequences, length of the direct repeats, marked identity, or similarity in the transposase sequence, as well as the chemistry of transposition [17]. As shown in Table 1, there was great IS diversity within *T. thermophilus* HB27 and HB8, including 11 different types of IS, 8 of which were specific to *Thermus* (ISTth1-8), and 2 types (IS1000A and its derivative IS1000B) belonging to the IS110 family. Additionally, *T. thermophilus* SG0.5 harbored different IS copies, the majority of them belonging to the heterogeneous IS4 family. Contrary to the normally low intra-species IS diversity [46], many *Thermus* isolates contained a great diversity of ISs belonging to different families (Table 2), suggesting coexistence in the same cell of different mechanisms of transposition and induction signaling. 

All ISTth types were of similar size (1214.10 ± 60.14 bp) and encoded transposases of approx. 360 amino acids in length. On average, conservation of the DDE motif (a common transposase active site) suggested a similar biochemical pathway of transposition for six of them (ISTth1, 2, 4, 5, 6, 7, and 8). By contrast, copies of IS1000A/B harbored DEDD motifs, thus supporting a different transposition mechanism. Interestingly, all typologies except ISTth4 were found in the laboratory model strain *T. thermophilus* HB27, whereas its related strain HB8 lacked ISTth2 and ISTth5 types (Table 1), and the NAR1 strain lacked ISTth2, ISTth5, and ISTth8.

Types ISTth4 and ISTth5 showed up to 65% identity, indicating their close relationship. An even greater degree of identity was found for IS1000A/IS1000B, which differed by only 14 bp. On the other hand, the greatest IS variability was observed in the 3′ ends, along the IS boundary (i.e., terminal IR regions) regardless of the genomic localization or bacterial strain. High variability was also observed in the target site duplication (TSD), which represented a unique hallmark for each IS. 

It is also interesting to note that, on average, the GC content of these complete and partial IS copies was similar to that of the host genome (mean 68.1%) regardless of the ISTth diversity, supporting a long-term co-evolution in these strains.

### 3.3. Evidence of ISTth Propagation to Other Microorganisms

We performed serial BLASTn analysis to examine if any of ISTth copies have contributed to horizontal gene transfer to other microorganisms. Within the *Thermus* genus, identical copies of ISTth3 were detected in plasmids of *T. aquaticus*, *T. oshimai*, and *T. parvatiensis*, clearly supporting the lateral exchange between these strains. Likewise, nine almost identical ISTth7 copies (1–3 bp mismatch) were found in isolates of *T. parvatiensis*, and several highly conserved ISTth4 copies (98% identical) were found in plasmids from a wide variety of *Thermus* members, suggesting its chief role in helping the spread of the *Thermus* pan-genome.

Beyond this putative transfer among *Thermus* strains, phylogenetically distant organisms, such as *Meiothermus* spp. may have acquired some ISTth copies (or vice versa). A total of 16 ISTth1 homologues (>86% sequence identity) were found in the genome of *M. silvanus* DSM9946, 12 of which were localized to the pMESIL plasmid, where they were renamed as ISMesil (ISfinder database). Furthermore, ISTth2, ISTth3, or ISTth8 were more than 50% identical to ISs present in other genera, including ISXaca1(*Xanthomonas campestris*), ISPlu4 (*Photorhabdus luminescens*), and ISDha5 (*Desulfitobacterium hafniense*). This great similarity may be a consequence of recent transfer events between these genera. Finally, copies of IS421 found in *T. thermophilus* SG0.5 showed 99% DNA sequence identity to *E. coli* IS186, supporting a transfer between these distant phylogenetic groups, despite their functionality at very different temperatures.

### 3.4. Distribution of Putatively Active vs. Inactive IS Copies across Selected T. thermophilus Genomes

**Figure 2:**
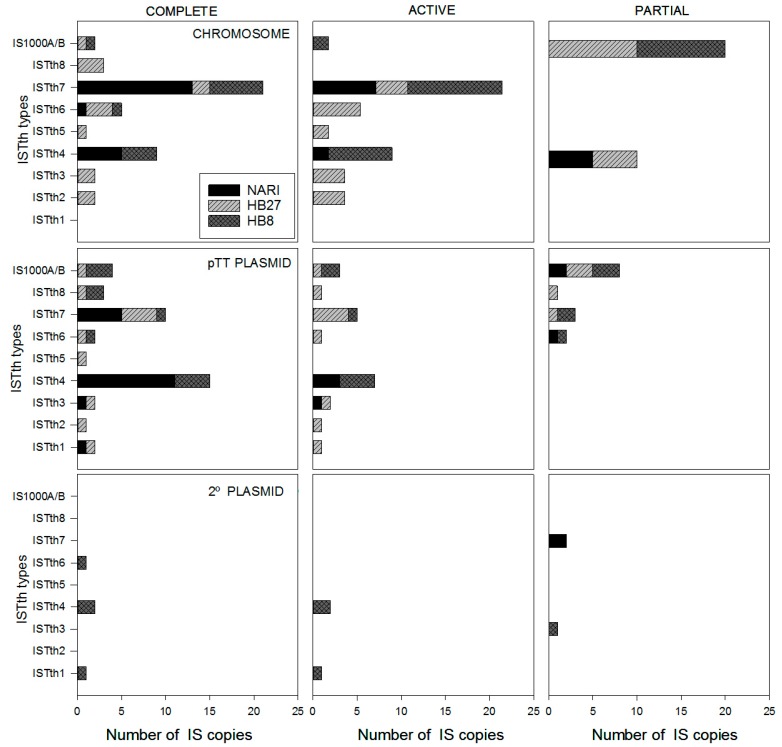
Abundance of insertion sequences across *Thermus thermophilus*. Number of full copies (complete), potentially active (middle column), and incomplete (partial) IS sequences found in the genomes of *T. thermophilus* HB27, HB8, and NARI strains. Sequences were localized to the chromosome (first row), pTT27 (HB27 and HB8), and M1 (pTT PLASMID; NAR1) megaplasmids (middle row), or in pVV8 (HB8) and M2 megaplasmid (NAR1) (2º PLASMID, third row).

The ISs present in aerobic (HB27 and HB8) and nitrate respiring (NAR1) strains of *T. thermophilus* are detailed in Figure 2. Excluding the 32 bp CRISPR repeats identical to IS1000A/B fragments described above, the *T. thermophilus* HB27 reference genome contained 25 complete copies of different ISs, of which 20 encode putatively active transposases, and 8 IS fragments. Figures were similar in *T. thermophilus* HB8, with 27 full copies, 21 of which were putatively active, and 9 IS fragments. It is worth mention that several copies of ISTth7 encoding two ORFs corresponding to the N- and C-terminal domains of the corresponding transposases were classified as active, according to the proposal of Gregory and Dhalberg [35]. At least one copy of every IS identified in each analyzed *T. thermophilus* strain was potentially active (Figure 2). ISTth7, which belongs to the IS5 family, was the most abundant in terms of number of copies in both strains (9 and 11 copies in HB27 and HB8, respectively), and also in the other sequenced *Thermus* spp. (Table 2). Also, numerous copies of ISTth4 were found in *T. thermophilus* HB8 [10] and NAR1 [19] strains, in contrast to its absence from HB27. On the contrary, ISTth1, ISTth2, and ISTth5 were the least abundant in these and all other published *Thermus* spp. genome sequences. In all the cases studied, the highest IS density and diversity was associated with the pTT27-related megaplasmids, with one member of each IS in both strains except from 4 copies of ISTth7 in HB27 pTT27, and 4 and 11 copies of ISTth4 in HB8 pTT27 and NAR1 M1 megaplasmid (Table 1).

Distribution of complete and partial copies of the ISs within the genome of these three strains revealed a higher concentration in megaplasmids (6.5%–7% of total sequence) than in the chromosome (0.8% of total sequence). This suggests a major role for the chromosome in coding for conserved core functions, which would act as a major counterselection factor if genes were interrupted by IS transposition. Also, our data are in agreement with the more flexible character of the megaplasmids, which encode adaptive and HGT-prone faculties that are only needed under specific conditions [30]. Our data are consistent with other observations of higher incidence of transposable elements within plasmids [47,48], and also that pTT27 harbors the highest plasticity with low synteny among strains [49]. 

**Figure 3:**
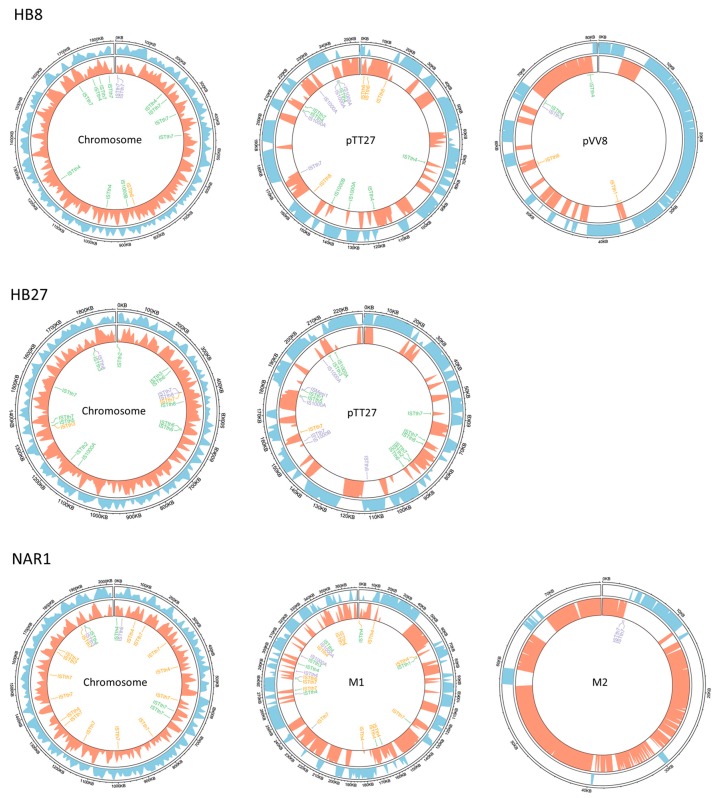
Location of ISs in the genomes of selected strains. The chromosome and megaplasmids (pTT, pTT27, PVV8, M1, and M2) of the *T. thermophilus* strains HB8, HB27, and NAR1 are represented as circles, with blue and orange shading indicating the coding densities of both DNA strands. Positions of the indicated IS are labeled with color codes indicating putatively active (green), inactive but complete (orange), or incomplete (purple). The replicons are drawn at different scales.

Even within the megaplasmids, there were regions which concentrated many complete and partial IS sequences (Figure 3), serving as hotspots for the integration of new genomic traits. One such example is the nitrate respiration island, whose insertion into pTT27 of strain HB27 after conjugative transfer from *T. thermophilus* NAR1 appears associated to a copy of ISTth7 [50].

### 3.5. Potentially Active Transposases

**Figure 4:**
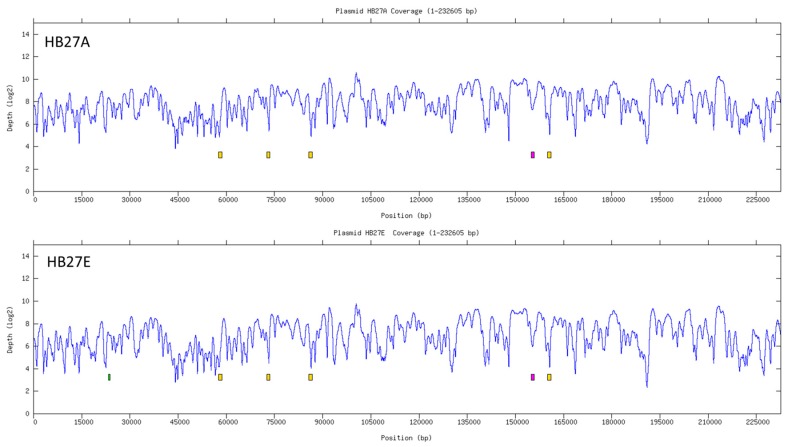
Identification of recent events of transposition by ISTth7 in the megaplasmid of *T. thermophilus* HB27. Coverage plot of HB27A (upper), HB27E (lower) reads plotted against the *T. thermophilus* HB27 reference pTT27 plasmid. Yellow boxes show the location of the ISTth7 transposase common between the reference HB27 genome and to the laboratory-adapted strains HB27A and HB27E. The pink box labels the position of an ISTth7 copy common to both HB27A and HB27E which is absent from the reference HB27, while the green box indicates the location of an ISTth7 copy specific to the HB27E strain.

The above data support the existence of a significant number of putatively functional ISs in the genomes of the three *T. thermophilus* strains analyzed. In order to further test this hypothesis in silico, we searched for recent integration events of ISTth7 in the genome of strain HB27. For this, two laboratory-adapted strains derived from *T. thermophilus* HB27 (strains HB27A and HB27E) were subjected to Illumina-based full genome sequencing (see Materials and Methods). In order to find recent movements of ISTth7, a procedure was followed in which we recruited those sequences of the HB27A and HB27E genomes, that overlapped the 3′ and 5′ extremes of ISTth7, searching for matches to the ISTth7 boundary sequences found in the reference HB27 genome. This comparison confirmed maintenance of 2 expected chromosomal copies (reference GenBank accession AE017221.1) and 4 copies expected in the megaplasmid (reference GenBank accession AE01722.2), and detected additional copies in both domesticated strains. Strain HB27E contained 7 additional copies in the chromosome and 2 additional copies in the megaplasmid (Table 3 and Table 4), of which 6 chromosomal copies and 1 megaplasmid copy were also found in HB27A (Figure 4).

Of the two additional ISTth7 copies found in HB27E compared to HB27A, one had already been identified in a previous study where it was shown to confer higher transformability. This was a consequence of the inactivation of the TTP0026 gene, which encodes a homologue to the eukaryotic Argonaute protein implicated in defense against invading DNA [51]. Its identification in the current study validated the procedure that was followed. In conclusion, our data demonstrate that ISTth7 is a very active insertion sequence in *T. thermophilus*, possibly playing a role in adaptation of this strain to laboratory growth conditions.

## 4. Discussion and Conclusions

Despite the extreme conditions and relatively isolated habitat of *Thermus* spp., several attributes leading to extraordinary genome plasticity enhance their evolutionary success. The first aspect of this plasticity is related to the presence (in most strains) of a constitutively expressed natural competence apparatus that allows cells to import environmental DNA (eDNA) at very high efficiencies, and also to incorporate genomic DNA from specific *Thermus* spp. donor strains (tDNA) through a novel conjugation-like mechanism (transjugation) [52]. In most cases, eDNA is degraded by different pathways, including restriction enzymes and nucleic acid interference, mediated either by diverse CRISPR/Cas systems or by Argonaute. However, tDNA acquired by transjugation has a greater chance to overcome these surveillance systems, which allows the recombination apparatus to incorporate new genes if they are bordered by sequences that are homologous to the host genome or to self-catalytic elements, such as ISs.

Recently, the dynamics of ISs within bacterial populations has come to the forefront, as the spread of pathogen-related genes and antibiotic resistances has been associated with transposable elements [13,24,53]. Although no actual transposons encoding selectable genes were detected in our analysis, horizontal gene transfer via ISs seems increased in several extreme environments, evidenced by the large numbers identified in the genomes of extremophilic microorganisms [23,25]. In particular, multiple reports on thermophiles have highlighted a surprisingly high diversity and number of ISs, suggesting an association with transfer of genes involved in thermophilic adaptation. For instance, *Deinococcus geothermalis* harbors more than 113 IS copies, classified into 6 types entailing up to 12% of its genome, whereas the closely related mesophilic *Deinococcus radiodurans* only presents 4 IS copies. Additionally, Blount and Grogan [54] reported an extensive diversity of ISs among isolated *Sulfolobus* populations (thermoacidophile) from a common sampling site, confirming that despite relative isolation, these thermophilic niches are notoriously rich in these elements. This surprising IS enrichment has been observed in other extremophiles, with similar figures described for the halophile *Haloarcula marismortui*, in which 20% of its pNG500 megaplasmid corresponded to IS DNA [55], and more than 169 IS sequences have been detected across the *Bacillus halodurans* genome [56]. Indeed, various metagenomic studies focused on extreme environments have revealed the preferred location of ISs to be within autonomous extrachromosomal elements [57]. This implies their role in the spread of adaptive genes through flexible genetic platforms like the megaplasmids present in many *Thermus* spp., which provides the cells a way to assess their selective value without risking essential chromosomal genes. Chromosomal IS copies may further facilitate the intra-genomic transfer of important traits, possibly explaining the diverse transposition frequencies, patterns, and abundance of IS scars and partial copies spread across thermophilic genomes [54].

Here, we have analyzed the abundance, diversity, and recent mobility events of IS elements in *Thermus* spp., with a special emphasis on the laboratory strains HB27, HB8, and NAR1. Our data support many of the aforementioned features common to many extremophiles: a high number of ISs with broad diversity and concentration of ISs within extrachromosomal elements, with densities of 69 IS copies/Mbp compared to 9 copies/Mbp found in the chromosome. In this regard, the significant increase in chromosomal ISTth7 copies found in the laboratory-adapted HB27 strains, compared to their parent, was very surprising. This could be explained by the dispensability of the targeted genes when grown in laboratory growth media, or the positive selection of these mutations under such conditions. A clear example of this would be selection of high transformation efficiency mutants (HB27E) by knockout of the *ago* gene in the megaplasmid (*TTP026::ISTth7*). Alternatively, it is also possible that the apparent increase in number of ISTth7 sequences found and the shared localization in both strains, despite their evolution in separate laboratories, is a consequence of contig collapse in the reference genome during assembly of the published genome.

Another consequence of the greater abundance of ISs in the megaplasmids is that it provides recombination sites for the insertion of DNA acquired via HGT. In this sense, it is relevant to note that genes associated with megaplasmids are tenfold more likely to be transferred by transjugation and integrated into other *T. thermophilus* recipient strains than chromosomal genes [57]. Thus, the presence of homologous ISs in donor and recipient strains may contribute to the integration step and, consequently, to the spread of adaptive traits such as nitrate respiration [50]. Also, ISs flanking genomic regions may mobilize groups of genes and generate phenotypic diversity, expanding genetic variability within the population and ultimately speeding its evolution. 

## Figures and Tables

**Table 1:** Presence of complete (C) and partial (P) IS sequences in the genomes of *T. thermophilus* strains HB27 (AE017221.1 and AE017222), HB8 (GCA_000091545.1 and AB677526), and NAR1 (PRJEB29203). Distribution among the chromosome (Ch) or the megaplasmids M1 and M2 from NAR1, pTT27 from HB27 and HB8, and pVV8 from HB8 is shown. Note that most partial copies of IS1000A/B actually correspond to CRISPR repeats (see the text for details).*

	NAR1						HB27				HB8					
	Ch		M1		M2		Ch		pTT27		Ch		pTT27		pVV8	
	C	P	C	P	C	P	C	P	C	P	C	P	C	P	C	P
**ISTth1**			1						1						1	
**ISTth2**							2		1							
**ISTth3**		1	1				2	1	1							1
**ISTth4**	5		11								4		4		2	
**ISTth5**							1		1							
**ISTth6**	1			1			3		1		1		1	1	1	
**ISTth7**	13		5			2	2	2	4	1	6	2	1	2		
**ISTth8**							3		1	1			2			
IS1000A/B*				2			1	8	1	34	1	14	3	39		

**Table 2:** Diversity of insertion sequences (ISs) across *Thermus* spp. Most relevant ISs found were characterized by the family and subgroup to which they belong, their main features (size, direct repeats (DRs), and number of ORFs), and their relative frequency among members of the genus *Thermus*. Frequency was graded as high (+++; present in more than 6 different isolates), moderate (++; present in 3–6 isolates) and low (+; present in 2 or fewer isolates). Presence across the genus was determined by BLASTn, with a threshold of E-value <10^−10^, query coverage >40%, and identity >70%.

Name	Family	Sub-Group	Size Range (bp)	DRs (bp)	N° ORF	Frequency in *Thermus* spp.
**ISTth1**	IS3	IS150	1200–1600	3–4	2	+
**ISTth2**	IS4	IS10	1200–1350	9	1	+
**ISTth3**	IS1634	IS4	1500–2000	5–6	1	+++
**ISTth4**	IS256	-	1200–1500	8–9	1	+++
**ISTth5**	IS256		1200–1500	0	1	+
**ISTth6**	IS630	-	1000–1400	2	1–2	++
**ISTth7**	IS5	ISH1	900–1150	8	1	+++
**ISTth8**	IS701	-	1400–1550	4	1	++
**IS1000A**	IS110	-	1136–1558	2	1	+++
**IS1000B**	IS110		954–1558	0	1	++
**IS421**	IS4	IS231	1450–5400	10–12	1	+

**Table 3:** Coordinate locations of ISTth7 soft-clipped reads in the chromosome of the reference strain *T. thermophilus* HB27 (GenBank accession AE017221.1).

Start	End	Found in Reference	Found in HB27A	Found in HB27E
259,293	259,789	NO	YES	YES
386,772	389,127	NO	YES	YES
562,354	562,716	NO	YES	YES
1,082,351	1,082,693	NO	NO	YES
1,133,885	1,134,345	NO	YES	YES
1,370,579	1,371,607	YES	YES	YES
1,533,343	1,534,271	YES	YES	YES
1,550,847	1,551,323	NO	YES	YES
1,655,564	1,655,957	NO	YES	YES

**Table 4:** Coordinates location of ISTth7 soft-clipped reads in the megaplasmid of the reference strain *T. thermophilus* HB27 (GenBank accession (AE017222).

Start	End	Found in Reference	Found in HB27A	Found in HB27E
23,402	23,834	NO	NO	YES
57,597	58,625	YES	YES	YES
72,589	73,617	YES	YES	YES
85,677	86,705	YES	YES	YES
154,975	155,865	NO	YES	YES
159,958	160,986	YES	YES	YES
